# Preoperative systemic inflammatory response index predicts long-term outcomes in type B aortic dissection after endovascular repair

**DOI:** 10.3389/fimmu.2022.992463

**Published:** 2022-09-28

**Authors:** Yufei Zhao, Xiang Hong, Xinsheng Xie, Daqiao Guo, Bin Chen, Weiguo Fu, Lixin Wang

**Affiliations:** ^1^ Department of Vascular Surgery, Zhongshan Hospital, Fudan University, Shanghai, China; ^2^ Department of Vascular Surgery, Zhongshan Hospital (Xiamen), Fudan University, Xiamen, China; ^3^ Institute of Vascular Surgery, Fudan University, Shanghai, China; ^4^ National Clinical Research Center for Interventional Medicine, Shanghai, China

**Keywords:** biomarkers, thoracic endovascular aortic repair, type B aortic dissection, systemic immune inflammation index (SII), systemic inflammatory response index (SIRI)

## Abstract

**Objectives:**

Inflammation is a hallmark of the initial development and progression of aortic dissection. This study aimed to investigate the value of preoperative inflammatory biomarkers in predicting aorta-related adverse events (AAEs) after thoracic endovascular aortic repair (TEVAR) for type B aortic dissection.

**Methods:**

We included all patients who underwent TEVAR for type B aortic dissection between November 2016 and November 2020 in this single-center, retrospective cohort study. Patients were divided into two groups: the AAEs group (n = 75) and the non-AAEs group (n = 126). Preoperative inflammatory biomarkers were recorded, including neutrophil-to-lymphocyte ratio (NLR), monocyte-to-lymphocyte ratio (MLR), platelet-to-lymphocyte ratio (PLR), systemic immune inflammation index (SII), and systemic inflammatory response index (SIRI). Patients were followed-up for the development of AAEs. Prediction accuracy of inflammatory biomarkers for AAEs were evaluated using the area under the receiver operating characteristic curves.

**Results:**

This study included 201 patients, of whom 80.0% were men, with a mean age of 59.1 ± 12.5 years. A total of 75 patients developed AAEs after TEVAR. The AUCs of NLR, MLR, PLR, SII, and SIRI for AAEs were.746,.782,.534,.625 and.807, respectively. Age and SIRI were independent risk factors for the AAEs after TEVAR (HR 3.264, p <.001; HR 4.281, p <.001, respectively). Survival analysis revealed significantly lower AAE-free status in patients with preoperative SIRI > = 4 (p <.001).

**Conclusion:**

Increased preoperative SIRI and age are independent risk factors for AAEs after TEVAR in type B aortic dissection.

## Graphical Abstract

This study investigated the value of preoperative inflammatory biomarkers NLR, MLR, PLR, SII, and SIRI in predicting aorta-related adverse events (AAEs) after thoracic endovascular aortic repair (TEVAR) for type B aortic dissection. The increased SIRI and age were independently associated with postoperative AAEs after TEVAR. Preoperative SIRI serves as an effective prognostic risk factor in patients with type B aortic dissection.

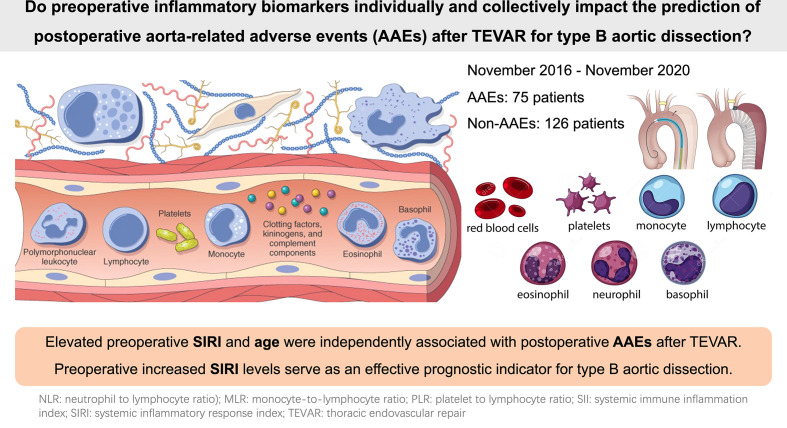

## Introduction

As we enter a new epoch of minimally invasive therapy, thoracic endovascular aortic repair (TEVAR) has become the first choice for complicated type B aortic dissection (TBAD) (1). However, a satisfactory aortic remodeling was limited to a subset of patients due to the postoperative adverse events following TEVAR.

The past decades have witnessed a growing number of biomarkers investigated to refine stratification of patients for diagnosis and prognosis prediction. While previous studies concerning the risk factors of postoperative complications after TEVAR focused mainly on anatomic and morphologic features and their changes during the follow-up based on imaging methods (2–5).

Although the pathogenesis of aortic dissection (AD) is not yet clear, accumulating clinical evidence indicate that an increased systemic inflammatory status is a crucial determinant of post-interventional outcomes after TEVAR for patients with AD (6–10). Several inflammation and immune-based prognostic scores were established to monitor the state of systemic inflammatory response, such as neutrophil-to-lymphocyte ratio (NLR), monocyte-to-lymphocyte ratio (MLR), and platelet-to-lymphocyte ratio (PLR), and were consequently proven to effectively predict the prognosis of various tumors (11–18) and cardiovascular diseases (19–22). In addition, the systemic immune-inflammation index (SII), which might more comprehensively reflect the balance of host inflammatory and immune status, was a promising independent predictive factor for prognosis of patients with hepatocellular carcinoma after surgery (23). The systemic inflammation response index (SIRI), firstly reported for its ability to predict the survival of patients with pancreatic adenocarcinoma after chemotherapy, was an amalgamated predictor to reflect innate and adaptive immunity in response to cancer (24).

Nonetheless, the risk stratification model of AD is yet to include inflammatory markers, of which the individual and combined impact on prediction of adverse events after TEVAR in TBAD remain unclear. This study aimed to elucidate the association between the preoperative inflammatory biomarkers (namely NLR, MLR, PLR, SII, and SIRI) and long-term clinical outcomes after TEVAR for TBAD.

## Materials and methods

### Study population

This study was approved by the Ethics Committee of Zhongshan Hospital of Fudan University (IRB number B2019-231R; December 18th, 2019). Informed consent was obtained from all the participants. We retrospectively collected and analyzed the clinical data of patients with type B aortic dissection who underwent surgery in the Zhongshan Hospital of Fudan University between November 2016 and November 2020. Patients were included if they were diagnosed with type B aortic dissection, which was confirmed through computed tomography angiography (CTA) and had signed informed consent for TEVAR, which was approved by institutional review board. Patients were excluded if they met any of the following criteria: (1) patients who had received open surgery due to Marfan syndrome or bicuspid aortic valve malformation; (2) patients had other conditions that affect the count of inflammatory cells such as active malignant tumors, acute infections, anti-inflammatory medication within the previous three months, hemopoietic system disorders, or autoimmune diseases; (3) patients whose preoperative laboratory data from primary or secondary centers were unavailable for review at the time of data collection; and (4) patients who were lost to follow-up postoperatively or not followed up to one year. Baseline clinical features, imaging results, surgical records, and clinical outcomes of the participants were obtained from their medical records.

### Exposure definition

The neutrophil-to-lymphocyte ratio (NLR) was defined as the number of neutrophils divided by the number of lymphocytes. The monocyte-to-lymphocyte ratio (MLR) was defined as the number of monocytes divided by the number of lymphocytes. The platelet-to-lymphocyte ratio (PLR) was defined as the number of platelets divided by the number of lymphocytes. The systemic immune-inflammation index (SII) was defined as the platelet count multiplied by the NLR. The systemic inflammation response index (SIRI) was defined as the monocyte count multiplied by the NLR. The first preoperative venous blood specimens, usually drawn within five days of surgery, were used.

### Outcome ascertainment

The endpoint of this study was the occurrence of postoperative AAEs after TEVAR during follow-up, which was diagnosed through computed tomography angiography and intraoperative digital subtraction angiography (DSA), including endoleak, distal stent-induced new entry (dSINE), retrograde type A AD (RTAD), distal aortic expansion, branch artery occlusion or stenosis, aortic rupture, and death. Two authors independently collected and reviewed the laboratory and clinical data and were blinded to the outcomes.

### Potential confounders

Overall, we considered potential confounders assessed before or at cohort entry; these variables included demographic characteristics, comorbidities, prescriptions, and aortic dissection-related variables. We included the following comorbidities measured at any time before cohort entry: hypertension, smoking and drinking histories, diabetes, angina pectoris, myocardial infarction, stroke (ischemic or hemorrhagic) or transient ischemic attack, chronic kidney disease, heart failure, peripheral artery disease. We also considered use of the following prescription drugs measured in the year before cohort entry: antihypertensive drugs, antiplatelets, anticoagulants, and statins. Last, the model included the following aortic dissection-related variables measured between the diagnosis date and cohort entry: intervention phase (acute, subacute, and chronic), the location of primary tear (Z3 or Z4), proximal landing zone, adjunctive procedure.

### Statistical analysis

We determined that a sample of 201 patients (75 in AAE group and 126 in non-AAE group) would provide a power of 95.2% to detect a difference in the proportion of patients with the primary endpoint at a two-sided significance level of 0.05. Continuous variables are presented as means with standard deviations (SDs) or as medians with interquartile ranges (IQRs). Comparisons between groups were made using the Student’s t-test or the Mann-Whitney U test for continuous variables and Pearson’s chi-square test or Fisher’s exact test for categorical variables. Normality was tested using the Kolmogorov-Smirnov test. Odds ratios (ORs) with 95% confidence intervals (CIs) were calculated through logistic regression. The receiver operating characteristic (ROC) curve was used to determine the optimal cut-off values of quantitative variables NLR, MLR, PLR, SII, and SIRI that predicted the occurrence of AAEs. Survival analysis was performed using Kaplan-Meier curves with the log-rank test to assess the differences in time-to-event endpoints. Univariable regression analysis was used to preliminarily analyze the risk factors for the occurrence of AAEs. Statistically significant variables (p <.2) on univariable Cox analysis were included in the multivariable Cox regression analysis to identify independent risk factors. Multiple imputation was used to account for the missing data (because of incomplete patient interviews, study dropouts, and deaths). Statistical significance was set at P <.05. Data analysis and visualization were performed using SPSS (IBM Corp., Armonk, NY, USA), GraphPad Prism 8.0 (GraphPad Software, San Diego, CA, USA), and R (R Foundation for Statistical Computing, Vienna, Austria).

### Sensitivity analysis

We conducted two sensitivity analyses to assess the robustness of our findings. First, we restricted the study population to patients with acute aortic dissection to minimize the potential difference between acute and chronic dissection. Second, we assessed the effect of variables with missing information (ie, intervention phase and the location of primary tear) by conducting multiple imputation with 10 imputations performed (**Supplementary Tables 3**, **4**).

This study was conducted in accordance with the STROBE guidelines and the principles of the Declaration of Helsinki.

## Results

### Demographic and baseline characteristics

**Figure 1:**
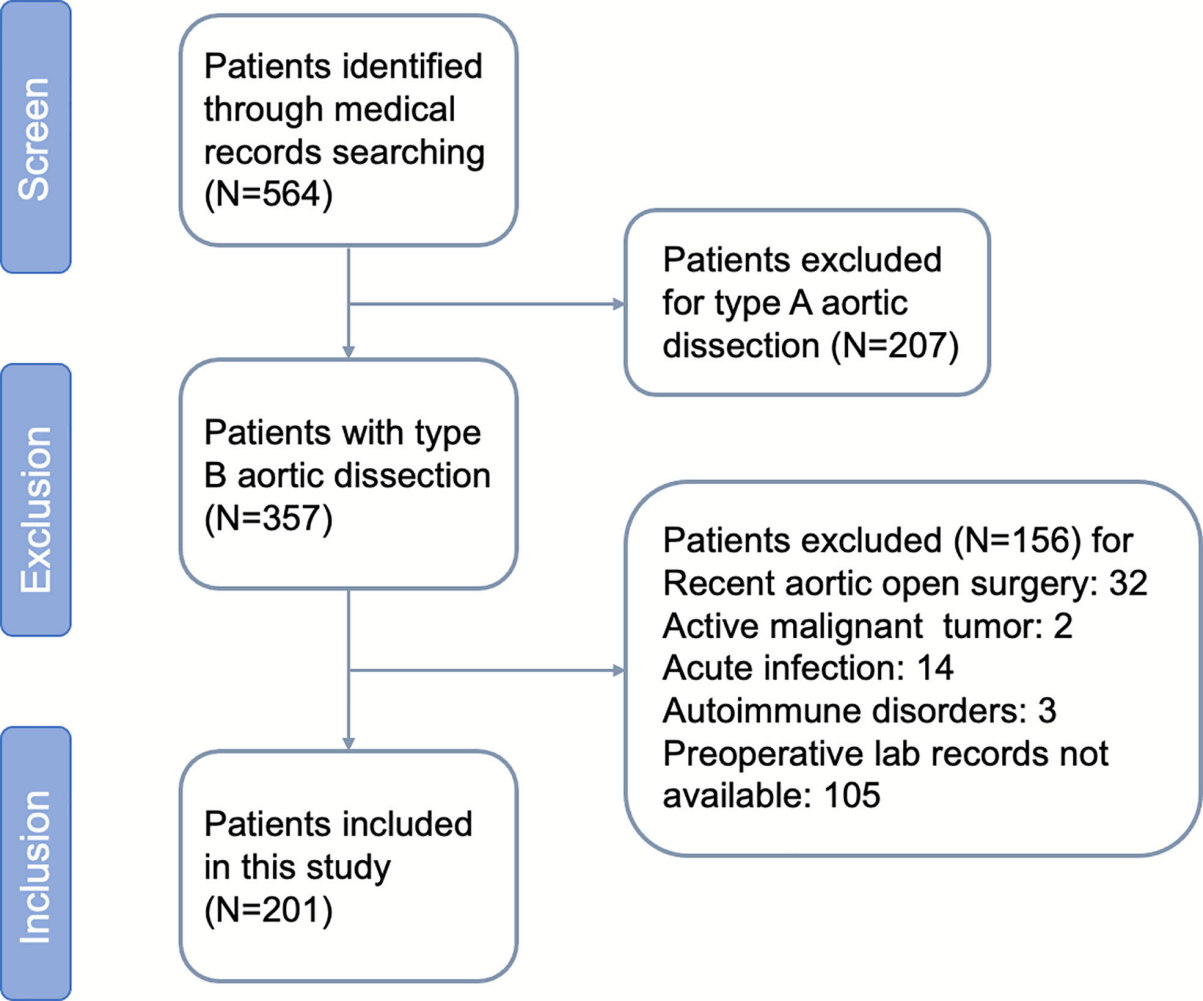
Flow diagram according to the Preferred Reporting Items.

A flowchart concerning patients included and excluded is shown (**Figure 1**). During the study period, 201 patients with aortic dissection remained after exclusions, of whom 80.0% were male, with a mean age of 59.1 ± 6.3 years. The median follow-up period was 31 months. A total of 75 patients developed AAEs after TEVAR, including 22 cases of endoleak (Ia, Ib, III), six of dSINE, one of RTAD, six of visceral artery stenosis or occlusion, four of left subclavian artery expansion or stenosis along with subclavian steal syndrome, two of thoracic aortic expansion, 33 distal aortic expansion resulting in distal aneurysm or dissection, and one death during hospitalization. The incidence of AAEs during the follow-up at six months, one year, three years and five years were 0.05%, 12.9%, 29.1%, and 39.8%, respectively.

The baseline characteristics of the AAE and non-AAE groups are shown in **Table 1**. Patients who developed AAEs after TEVAR had a mean age of 62.0 ± 4.4 years, which was higher than that of those without AAEs (p <.001). There were no statistically significant differences between male and female patients (p = .625). A history of hypertension, which was the most common risk factor for AD (75.1%), diabetes mellitus, coronary artery disease, acute myocardial infarction, stroke, chronic kidney disease, smoking, drinking, lipid-lowering drugs (such as statins), antiplatelet or anticoagulant drugs did not differ significantly between the AAE and non-AAE groups (p >.05). Similarly, intervention phase, the location of primary tear, proximal landing zone (to the left subclavian artery), and adjunctive procedure (such as *in situ* fenestration and chimney) showed no significant differences between the AAE and non-AAE groups (p >.05).

**Table 1:** Demographic and baseline characteristics.

	General population (n=201)	AAE group (n=75)	non-AAE group (n=126)	P-value
**Demographics**				
**Age**	59.11 ± 12.46	63.11 ± 11.13	57.59 ± 11.47	0.001 †
				
**Gender**				0.625 ‡
**Male**	162	60 (80.0)	102 (81.0)	
**Female**	39	16 (21.3)	23 (18.3)	
**Risk factors and comorbidities**				
**Hypertension**	151 (75.1)	61 (81.3)	90(71.4)	0.067 ‡
**Smoking**	54 (26.9)	21 (28.0)	33 (26.2)	0.780 ‡
**Alcohol**	23 (11.4)	5 (6.7)	18 (14.3)	0.101 ‡
**Diabetes**	18 (89.6)	5 (6.7)	13 (10.3)	0.381 ‡
**History of CAD**	10 (49.8)	2 (2.7)	8 (6.4)	0.246 ‡
**History of AMI**	1 (0.5)	0	1 (0.8)	0.194 ‡
**History of CVD**	6 (29.9)	1 (1.3)	5 (4.0)	0.288 ‡
**History of CKD**	6 (29.9)	2 (2.7)	4 (3.2)	0.838 ‡
**Medication on admission**				
**Antiplatelet**	9 (4.5)	3 (4.0)	6 (4.8)	0.801 ‡
**Anticoagulant**	1 (0.5)	0	1 (0.8)	0.194 ‡
**Statin**	6 (2.9)	2 (2.7)	4 (3.2)	0.838 ‡
**Phase**	175 (80.6)			0.221 ‡
**Acute**	131 (65.2)	47 (62.7)	84 (66.7)	
**Subacute**	30 (14.9)	12 (16.0)	18 (14.3)	
**chronic**	14 (7.0)	2 (2.7)	12 (9.5)	
**Location of primary intimal tear**				0.592 ‡
**Z3**	127 (63.2)	39 (52.0)	88 (69.8)	
**Z4**	9 (4.5)	2 (2.7)	7 (5.6)	
**The length of proximal landing zone (mm)**	1.72	1.67	1.74	0.400 †
**Length of stent-graft (mm)**	184.3	186	183.5	0.605 †
**Adjunctive procedures**	13 (6.5)	3 (4.0)	10 (7.9)	0.272 ‡
**Preoperative hematological parameters**				
**Serum creatinine (>115μmol/L)**	43 (21.4)	16 (21.3)	27 (21.4)	0.987 ‡
**eGFR (<100 ml/min/1.73m^2^)**	149 (74.1)	57 (76.0)	92 (73.0)	0.640 ‡
**Cholesterol (mmol/L)**	17 (8.5)	7 (9.3)	10 (7.9)	0.537 ‡
**Triglycerides (mmol/L)**	33 (16.4)	7 (9.3)	26 (20.6)	0.068 ‡
**LDL (mmol/L)**	50 (24.9)	20 (26.7)	30 (23.8)	0.714 ‡
**HDL (mmol/L)**	42 (20.9)	10 (13.3)	32 (25.4)	0.084 ‡
**Albumin (<35g/L)**	75 (37.3)	30 (40.0)	45 (35.7)	0.055 ‡
**WBC (×10^9/^L)**	75 (37.3)	26 (34.7)	49 (38.9)	0.549 †
**Neutrophil (×10^9/^L)**	74 (36.8)	32 (42.7)	42 (33.3)	0.001 †
**Monocyte (×10^9/^L)**	75 (37.3)	19 (25.3)	56 (44.4)	0.002 †
**Lymphocyte (×10^9/^L)**	69 (34.3)	32 (42.7)	37 (29.4)	0.001 †
**Platelet (×10^9/^L)**	40 (19.9)	11 (14.7)	29 (23.0)	0.004 †
**NLR (>5.105)**	55 (27.4)	25 (33.3)	30 (23.8)	< 0.001 ‡
**MLR (>0.675)**	74 (36.8)	23 (30.7)	51 (40.5)	0.163 ‡
**PLR (>127.985)**	61 (30.3)	44 (58.7)	17 (13.5)	< 0.001 ‡
**SII (>596.910)**	152 (75.6)	67 (89.3)	85 (67.5)	< 0.001 ‡
**SIRI (>3.990)**	76 (37.8)	53 (70.7)	23 (18.3)	< 0.001 ‡

SBP, systolic blood pressure; DBP, diastolic blood pressure; CAD, coronary artery disease; AMI, acute myocardial ischemia; CVD, cerebrovascular disease; CKD, chronic kidney disease; eGFR, estimated glomerular filtration rate; LDL, low-density lipoprotein; HDL, high-density lipoprotein; WBC, white blood cell; NLR, neutrophil to lymphocyte ratio; MLR, monocyte to lymphocyte ratio; PLR, platelet to lymphocyte ratio; SII, the ratio of platelet count multiply neutrophil count to lymphocyte count; SIRI, the ratio of monocyte count multiply neutrophil count to lymphocyte count.

Continuous variables are presented as means with standard deviations.

^†^t test or Mann-Whitney test.

^‡^Pearson chi-square test or Fisher’s exact test.

Compared to the non-AAE group, the AAE group exhibited a significant increase in neutrophil and monocyte counts, but a lower lymphocyte count and platelet count (P = .001,.002,.001,.004, respectively). Nonetheless, no significant difference was observed between the two groups in terms of white leukocyte, albumin, LDL, HDL, cholesterol, triglycerides, and serum creatinine (P = .549,.055,.714,.084,.537,.068, and.987, respectively).

The preoperative NLR, PLR, SII and SIRI were significantly different between the AAE and non-AAE groups (p <.001,.001,.001, and.001, respectively), while MLR did not differ significantly between both groups (p = .163).

### Preoperative inflammatory biomarkers and AAEs

**Figure 2:**
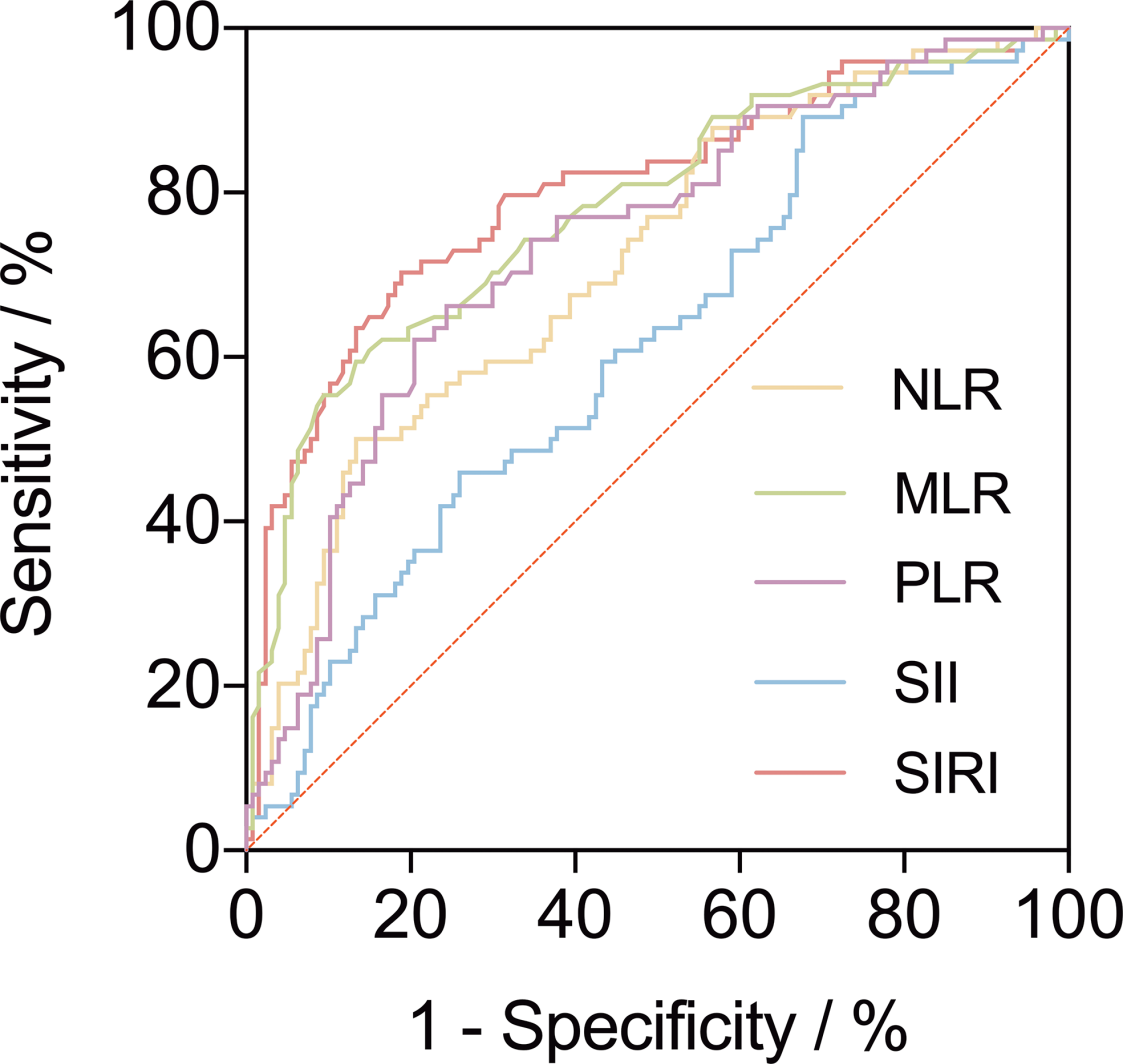
A receiver operating characteristic curve (ROC) to explore the value of pre-operative inflammatory biomarkers to identify aorta-related adverse events (AAEs) after thoracic endovascular repair. The area under the ROC curve of neutrophil-to-lymphocyte ratio, monocyte-to-lymphocyte ratio, platelet-to-lymphocyte ratio, systemic immune inflammation index and systemic inflammatory response index were 0.746, 0.782, 0.534, 0.625, and 0.807, respectively.

A ROC curve was used to explore the relationship and determine the optimal cut-off value between preoperative inflammatory biomarkers and AAEs after TEVAR (**Figure 2**). The area under the ROC curve of NLR, MLR, PLR, SII, and SIRI were.746,.782,.534,.625, and.807, respectively (**Table 2**). The ROC analysis showed that SII had the highest sensitivity of 89.3%, while MLR had the highest specificity of 86.5%.

**Table 2:** Receiver operating characteristic curve analysis.

AAEs	cut-off value	AUC	sensitivity	specificity
NLR	5.105	0.746	0.667	0.762
MLR	0.675	0.782	0.587	0.865
PLR	127.985	0.534	0.693	0.405
SII	596.91	0.625	0.893	0.325
SIRI	3.99	0.807	0.707	0.817

NLR, Neutrophil-to-lymphocyte ratio; MLR, Monocyte-to-lymphocyte ratio; PLR, Platelet-to-lymphocyte ratio; SII, Systemic immune inflammation index; SIRI, Systemic inflammatory response index; OR, odds ratio; CI, confidence interval; AUC, the area under the receiver operating characteristic curve.

Univariable logistic regression analysis showed that age, monocyte, lymphocyte, MLR, SII, and SIRI were associated with AAEs (**Supplementary Table 1**). Since we were more concerned about the relationship between inflammatory factors and the prognosis of type B AD, we also included NLR and PLR in the multivariable regression model. The results showed that age, MLR, and SIRI were independent risk factors for AAEs (OR 6.067, p <.001; OR 3.519, p <.001; OR 6.583, p <.001, respectively) (**Supplementary Table 2**).

Univariable and multivariable Cox proportional hazard regression analyses are illustrated in **Table 3**. Patients aged ≥ 55, with NLR ≥ 5.105, SII ≥ 127.985, SIRI ≥ 3.990 and underwent intervention in acute phase were associated with AAEs on univariable Cox regression. The multivariable Cox proportional hazard regression analysis finally showed that age and SIRI ≥ 3.990 were independent risk factors for AAEs (HR 3.264, p <.001; and HR 4.281, p <.001, respectively).

**Table 3:** Cox regression analysis.

	Univariable Cox regression		Multivariable Cox regression	
	HR (95% CI)	P-value	HR (95% CI)	P-value
**Age (>54.5)**	3.064	<0.001	3.264 (1.690-6.305)	< 0.001
**NLR (>5.105)**	3.233	<0.001	–	0.316
**MLR (>0.675)**	1.193	0.483	–	–
**PLR (>127.985)**	0.458	0.087	–	–
**SII (>596.910)**	2.675	0.006	–	0.685
**SIRI (>3.990)**	4.362	<0.001	4.281 (2.458-7.455)	< 0.001
**Acute/Chronic**	4.427	0.035	–	0.137
**Location of primary intimal tear**	7.052	0.632	–	–
**The length of proximal landing zone (1.45cm)**	13.435	0.008	–	0.786
**Adjunctive procedures**	3.683	0.055	–	0.146

NLR, Neutrophil-to-lymphocyte ratio; MLR, Monocyte-to-lymphocyte ratio; PLR, Platelet-to-lymphocyte ratio; SII, Systemic immune inflammation index; SIRI, Systemic inflammatory response.

**Figure 3:**
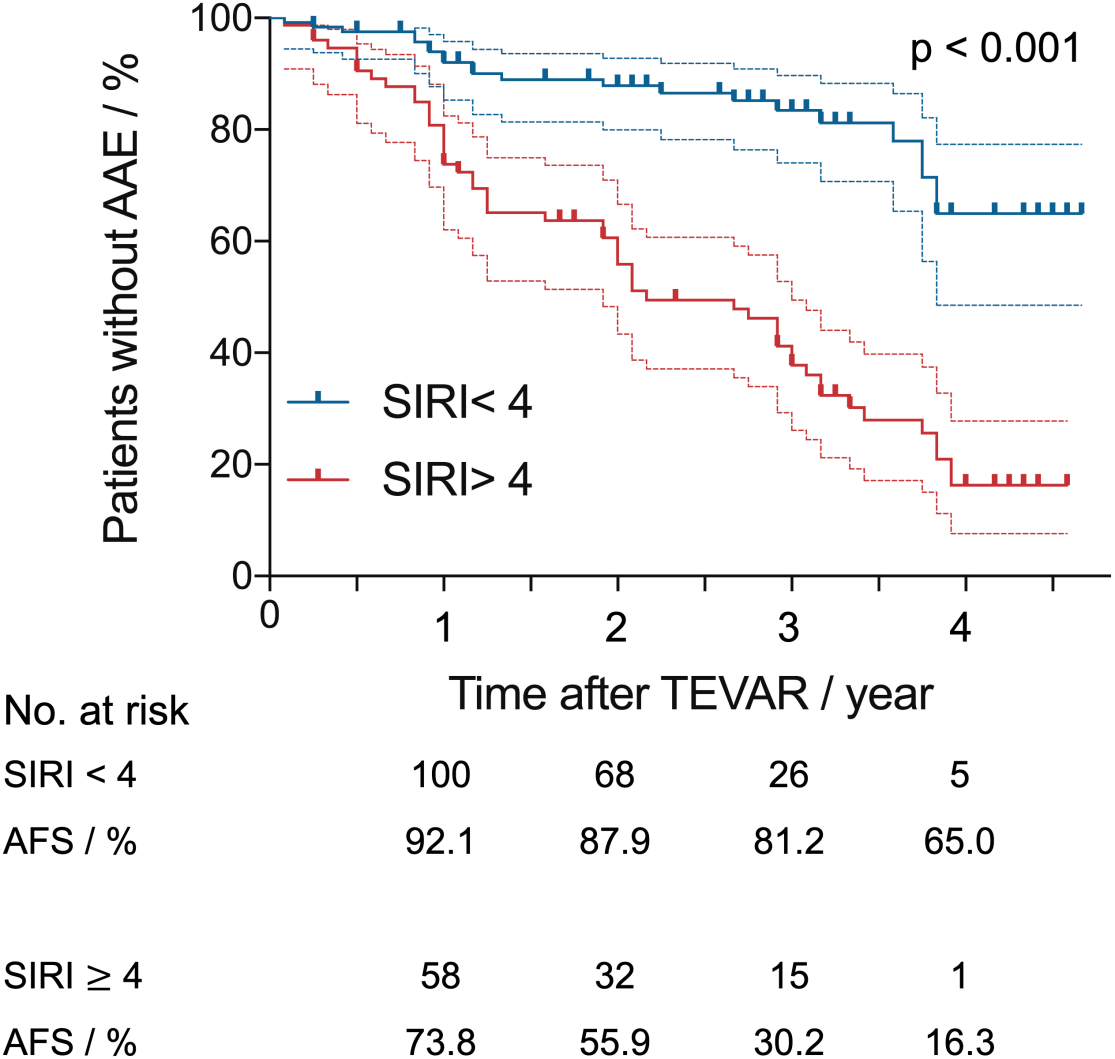
Kaplan–Meier aorta-related adverse events-free survival (AFS) curves after thoracic endovascular repair with systemic inflammatory response index (SIRI) of > 4 versus < 4. Dashed lines indicate the upper and lower limit for 95% CI.

Kaplan–Meier AAE-free survival (AFS) curves for SIRI are shown in **Figure 3**. The median survival for the preoperative SIRI ≥ 4 group was 21.9 ± 14.5 months, and the median survival for the preoperative SIRI < 4 group was 29.8 ± 15.9 months. Survival analysis revealed significantly lower six-month, one-year, three-year and five-year AFS in patients with a preoperative SIRI ≥ 4 than in those with a SIRI < 4 (log-rank test, p <.001).

## Discussion

In this cohort of patients with type B AD, a novel prognostic score, SIRI, constructed based on neutrophil, monocyte, and lymphocyte counts was found as an independent risk factor for AAEs after TEVAR with a prediction accuracy of 0.80. The risk of patients with a SIRI greater than 4 of developing adverse events after TEVAR was approximately increased by a factor of 4.3 in comparison to patients with a SIRI below 4.

A majority of studies have confirmed that NLR was elevated in patients with more advanced or aggressive cancer with an independent prognostic value in unselected cohorts, as evidenced by increased tumor stage, nodal stage, and metastatic lesions (25). A retrospective single-center analysis of 682 patients revealed NLR > 3.5 and MLR > 0.2 can be regarded as significant predictors of all-cause long-term mortality after Off-Pump coronary artery bypass grafting revascularization (26). The NLR combined with PLR can predict the prognosis of patients with acute myocardial infarction (27). Moreover, the NLR was proven to independently predict major adverse cardiovascular events risk and all-cause mortality, which was reduced by interleukin-1β blockade with canakinumab in five randomized trials (28). Likewise, the NLR was demonstrated to predict in-hospital mortality in patients with acute type A aortic dissection (TAAD), however, with discrepant cut-off values of 8.51 (29), 8.78 (30), and 6.0 (31) reported in different studies.

Although as an easily available and widely acceptable inflammatory biomarker, the NLR exhibits a strong ability to identify patients with a higher risk of poor outcomes, representing both innate and adaptive immune responses to the pathogenesis of AD, the role of monocytes and macrophages, a fundamental component of non-specific immunity, cannot be underappreciated or neglected. Most of the evidence points to an important contribution of monocyte­derived macrophages to the predominant infiltration and accumulation of macrophages in regions of medial disruption toward the adventitial side (32–34). Macrophages play a role in striking the balance between the promotion and resolution of the inflammatory response, and the activities of matrix metalloproteinases (MMPs) and tissue inhibitors of metalloproteinases, resulting in extracellular matrix remodeling and reparative tissue healing (35–37). Therefore, the SIRI represented an integrated indicator that comprehensively reflect the status of the systemic inflammation and immune response. Firstly reported by Qi et al. for its ability to predict the survival of patients with pancreatic adenocarcinoma after gemcitabine-based chemotherapy (24), the SIRI could also be a novel promising inflammatory biomarker for predicting all-cause mortality in elderly patients with heart failure (38).

The current treatment strategy of AD often utilizes the one-size-fits-most approach, where many patients would probably be prescribed the same as anyone else, without taking into account family history, age, sex, anatomical and morphological features of aorta, systemic and local inflammatory status. While the SIRI may change the pattern for its ability to assist preoperative risk stratification by identifying patients at higher risk of developing AAEs. When facing a patient with elevated SIRI before surgery, a strengthened perioperative management and a more stringent follow-up strategy for high-risk patients are recommended, such as including inflammatory markers tests into routine follow-up, shorten follow up interval and extending follow-up term.

Increasing evidence in mouse models, supported by human data, has corroborated the importance of systemic and local inflammation, and the efficacy of anti-inflammatory treatment during the remission of AD. Targeting the nucleotide-binding oligomerization domain–like receptor pyrin domain containing 3 (NLRP3)–caspase-1 inflammasome cascade with its inhibitor was verified to prevent AD through mitigating smooth muscle cell contractile protein degradation and extracellular matrix destruction (39, 40). In addition, the downstream effector of inflammasome, interleukin-1β was elevated in aortic tissue of AD, while interleukin-1β blocking could delay the progression through inhibiting the expression of MMP-2 and MMP-9 and the breakage of elastin fibers (41, 42).

The proof of concept that targeting inflammation reduces cardiovascular events has highlighted the need to develop new immunotherapy to treat patients with atherosclerotic cardiovascular disease (43). Likewise, considering the prognostic value of inflammatory biomarkers established based on clinical evidence, and the potential benefit of anti-inflammatory therapy for patients with AD as indicated in animal experiments, identifying novel strategies that harness anti-inflammatory treatment or immunotherapy in all candidate patients or to be tailored to specific groups of patients with AD may be necessary, to some extent, urgent in the clinical practice.

As with all observational studies, ours had some limitations. First, problems inherent in a single-center study may enable the results and the value possibly skewed, which therefore, warrants further evaluation of external validity in a large multi-center prospective cohort study. Second, determining the pathogenic mechanism by which increased SIRI levels indicate a poor outcome is beyond the scope of this study, and intervention study may provide further insight.

## Conclusion

An elevated preoperative systemic inflammatory response index (SIRI) and age are independent risk factors for aorta-related adverse events after thoracic endovascular aortic repair in type B aortic dissection. The SIRI, an easily measured inflammation and immune-based score, was introduced in prognosis evaluation of type B aortic dissection, beyond which the risk of aorta-related adverse events more than quadrupled.

## Data availability statement

The original contributions presented in the study are included in the article/**Supplementary Material**. Further inquiries can be directed to the corresponding authors.

## Ethics statement

The studies involving human participants were reviewed and approved by B2019-231R. The patients/participants provided their written informed consent to participate in this study. Written informed consent was obtained from the individual(s) for the publication of any potentially identifiable images or data included in this article.

## Author contributions

Data collection was conducted by YZ, XX, and XH; statistical analysis was performed by YZ and XX; study design was proposed and manuscript writing were performed by YZ; LW, WF, DG, and BC provided guidance and suggestion of statistical analysis, data interpretation, and discussion revision. All authors contributed to the article and approved the submitted version.

## Funding

This work was supported by the National Natural Science Foundation of China (grant number: 81970412), Science and Technology Innovation Plan of Shanghai Science and Technology Commission (grant number: 18441902400), Xiamen Municipal Health Science and Technology Program Fund (grant number: 3502Z20194034), and Xiamen Medical and health Guidance project (grant number: 3502220214201088).

## Conflict of interest

The authors declare that the research was conducted in the absence of any commercial or financial relationships that could be construed as a potential conflict of interest.

## Publisher’s note

All claims expressed in this article are solely those of the authors and do not necessarily represent those of their affiliated organizations, or those of the publisher, the editors and the reviewers. Any product that may be evaluated in this article, or claim that may be made by its manufacturer, is not guaranteed or endorsed by the publisher.

## Abbreviation

AD, Aortic dissection; AUC, Area under the ROC curve; AAEs, Aorta-related adverse events; dSINE, Distal stent-induced new entry; IRAD, International Registry of acute Aortic Dissection; NLR, Neutrophil-to-lymphocyte ratio; MLR, Monocyte-to-lymphocyte ratio; PLR, Platelet-to-lymphocyte ratio; ROC, Receiver operating characteristic curve; RTAD, Retrograde type A aortic dissection; SII, Systemic immune inflammation index; SIRI, Systemic inflammatory response index; TAAD, type A aortic dissection; TBAD, type B aortic dissection; TEVAR, Thoracic endovascular aortic repair.
