# Supplementary material for: Preoperative systemic inflammatory response index predicts long-term outcomes in type B aortic dissection after endovascular repair
Source: Front Immunol. 2022 Sep 28;13:992463. doi: 10.3389/fimmu.2022.992463 (PMC9554789; doi:10.3389/fimmu.2022.992463)
Supplement: Supplementary file 1 [file DataSheet_1.docx]

| Supplementary Table 1. Univariable logistic regression analysis | | |
| --- | --- | --- |
|  | OR (95% CI) | P-value |
| Demographics |  |  |
| Age | 6.33 (2.84-9.82) | < 0.001 † |
| Gender | 0.85 (0.41-1.73) | 0.645 ‡ |
|  |  |  |
| Risk factors and comorbidities |  |  |
| SBP | 1.19 (0.67-2.13) | 0.548 ‡ |
| DBP | 1.75 (0.98-3.13) | 0.058 ‡ |
| Smoking | 1.10 (0.56-2.08) | 0.780 ‡ |
| Alcohol | 0.43 (0.15-1.21) | 0.109 ‡ |
| Diabetes | 0.62 (0.21-1.82) | 0.384 ‡ |
| History of CAD | 0.40 (0.08-1.96) | 0.260 ‡ |
| Histoty of AMI | - | - |
| History of Stroke | 0.33 (0.04-2.85) | 0.312 ‡ |
| History of CKD | 0.84 (0.15-4.68) | 0.838 ‡ |
|  |  |  |
| Medication on admission |  |  |
| Statin | 0.84 (0.15-4.68) | 0.836 ‡ |
| Antiplatelet | 0.83 (0.20-3.44) | 0.801 ‡ |
| Anticoagulant | - | - |
|  |  |  |
| Preoperative hematological parameters |  |  |
| Serum creatinine (>115μmol/L) | 0.99 (0.49-2.00) | 0.987 ‡ |
| eGFR (<100 ml/min/1.73m^2^) |  | 0.768 ‡ |
| Cholesterol (mmol/L) | 1.38 (0.49-3.89) | 0.538 ‡ |
| Triglycerides (mmol/L) | 0.43 (0.17-1.08) | 0.073 ‡ |
| LDL (mmol/L) | 0.88 (0.44-1.76) | 0.714 ‡ |
| HDL (mmol/L) | 0.49 (0.22-1.11) | 0.088 ‡ |
| ALB (<35 g/L) | 0.78 (0.44-1.40) | 0.405 ‡ |
| WBC (×10^9^ /L) | 0.83 (0.46-1.51) | 0.550 † |
| Neutrophil (×10^9^ /L) | 1.49 (0.83-2.68) | 0.186 † |
| Monocyte (×10^9^ /L) | 0.37 (0.20-0.70) | 0.002 † |
| Lymphocyte (×10^9^ /L) | 2.14 (1.17-3.92) | 0.014 † |
| Platelet (×10^9^ /L) | 0.34 (0.12-1.40) | 0.546 † |
| NLR (>5.105) | 1.60 (0.85-3.01) | 0.145 ‡ |
| MLR (>0.675) | 9.10 (4.58- 18.10) | < 0.001 ‡ |
| PLR (>127.985) | 0.65 (0.36-1.20) | 0.164 ‡ |
| SII (>596.910) | 4.04 (1.78-9.20) | 0.001 ‡ |
| SIRI (>3.990) | 10.79 (5.51-21.12) | < 0.001 ‡ |

SBP = systolic blood pressure; DBP = diastolic blood pressure; CAD = coronary artery disease; AMI = acute myocardial ischemia; CVD = cerebrovascular disease; CKD= chronic kidney disease; eGFR = estimated glomerular ﬁltration rate; LDL = low-density lipoprotein; HDL = high-density lipoprotein; WBC = white blood cell; NLR = Neutrophil-to-lymphocyte ratio; MLR = Monocyte-to-lymphocyte ratio; PLR = Platelet-to-lymphocyte ratio; SII = Systemic immune inflammation index; SIRI = Systemic inflammatory response index; OR = odds ratio; CI= conﬁdence interval.

Continuous variables are presented as means with standard deviations.

† t test or Mann-Whitney test

‡ Pearson chi-square test or Fisher's exact test

| Supplementary Table 2. Multivariable logistic regression analysis | | |
| --- | --- | --- |
|  | OR (95% CI) | P-value |
| Age (>54.5) | 6.07 (2.58-14.25) | < 0.001 |
| NLR (>5.105) | - | 0.445 |
| MLR (>0.675) | 3.52 (1.45-8.53) | < 0.001 |
| PLR (>127.985) | - | 0.686 |
| SII (>596.910) | - | 0.806 |
| SIRI (>3.990) | 6.58 (2.76-15.68) | < 0.001 |

NLR = Neutrophil-to-lymphocyte ratio; MLR = Monocyte-to-lymphocyte ratio; PLR = Platelet-to-lymphocyte ratio; SII = Systemic immune inflammation index; SIRI = Systemic inflammatory response index; OR = odds ratio; CI= conﬁdence interval.

**Supplementary Table 3. Multivariable Cox regression: acute dissection**

|  | HR (95% CI) | P-value |
| --- | --- | --- |
| Age | - | 0.495 |
| NLR | - | 0.290 |
| MLR | - | 0.276 |
| PLR | - | 0.809 |
| SII | - | 0.418 |
| SIRI | 1.70(1.02-2.83) | 0.042 |

NLR = Neutrophil-to-lymphocyte ratio; MLR = Monocyte-to-lymphocyte ratio; PLR = Platelet-to-lymphocyte ratio; SII = Systemic immune inflammation index; SIRI = Systemic inflammatory response index; OR = odds ratio; CI= conﬁdence interval.

**Supplementary Table 4. Multivariable Cox regression: multiple imputation**

|  | HR (95% CI) | P-value |
| --- | --- | --- |
| Age | 3.36 (2.34-4.56) | 0.002 |
| NLR | - | 0.787 |
| MLR | - | 0.066 |
| PLR | - | 0.246 |
| SII | - | 0.829 |
| SIRI | 2.83 (1.96-3.35) | 0.006 |

NLR = Neutrophil-to-lymphocyte ratio; MLR = Monocyte-to-lymphocyte ratio; PLR = Platelet-to-lymphocyte ratio; SII = Systemic immune inflammation index; SIRI = Systemic inflammatory response index; OR = odds ratio; CI= conﬁdence interval.

**Supplementary Table 5: Lasso regression**

|  | Regression coefficient |
| --- | --- |
| Age | 0.040753513 |
| NLR | 0.096684089 |
| MLR | 1.414029061 |
| PLR | -0.001685885 |
| SII | - |
| SIRI | 0.153942063 |
